# Pellagra Forum

**Published:** 1916-09

**Authors:** W. L. Lee

**Affiliations:** Route 5, Hattiesburg, Miss.; Hot Springs, Ark.


### PELLAGRA FORUM.

DR. E. H. MARTIN, EDITOR, HOT SPRINGS, ARK.

Address all communications on this subject to Dr. Martin.

---

*Pellagra Editor, Texas Medical Journal:*

After reading the editor's comments on Dr. Perdue's able article on pellagra, I just could not help writing a letter on the subject. I have been investigating pellagra for six years or more; have followed out theory after theory, and the most nonsensical one, I think, is that of Dr. Goldberger. He simply starved a lot of convicts at the Jackson, Miss., convict farm, reduced them down to the lowest point of resistance. If the germ of tuberculosis had been lying dormant in any of them, he would have produced a case of tuberculosis. Let it be remembered that these convicts were drinking water daily that was loaded with silica, and when reduced by starvation to where their systems could not longer throw it off, the silica got in its work. He did not prove anything, only that a starved man could not resist disease like a well fed, healthy man can. I have been in touch with Dr. Perdue for more than a year; have tried out his treatment in a number of cases, and successfully with all who reached me before the brain and nervous system was wrecked beyond human help. The theory advocated by Dr. Perdue is no longer a theory, but an established fact proven beyond a question. Yes, geological conditions are old, and pellagra is new. That's a fact, and it is also a fact that we have had a surface soil of lime, that kept silica precipitated, and we got the water free from silica. We no longer have the surface soil of lime, and we are getting the colodial silica, hence pellagra. Colodial silica is the cause of pellagra, producing an intoxication, chronic acidosis. If any physician will start out to find the truth as to pellagra, regardless of Dr. Goldberger, or anyone else, and make an honest investigation of the silica theory, he just can't help but be convinced as to its correctness. Then try out the treatment faithfully, and he will be convinced again. Write Dr. Perdue on any point. You will find him to be not only a very able man, but a very courteous gentleman, always ready to give you any information on the subject.

W. L. LEE, M. D.,
Route 5, Hattiesburg, Miss.

